# ChemicalTagger: A tool for semantic text-mining in chemistry

**DOI:** 10.1186/1758-2946-3-17

**Published:** 2011-05-16

**Authors:** Lezan Hawizy, David M Jessop, Nico Adams, Peter Murray-Rust

**Affiliations:** 1 Unilever Centre for Molecular Science Informatics, Department of Chemistry, Lensfield Road, Cambridge, CB2 1EW, UK; 2 European Bioinformatics Institute, Wellcome Trust Genome Campus, Hinxton, Cambridge, CB10 1SD, UK

## Abstract

**Background:**

The primary method for scientific communication is in the form of published scientific articles and theses which use natural language combined with domain-specific terminology. As such, they contain free owing unstructured text. Given the usefulness of data extraction from unstructured literature, we aim to show how this can be achieved for the discipline of chemistry. The highly formulaic style of writing most chemists adopt make their contributions well suited to high-throughput Natural Language Processing (NLP) approaches.

**Results:**

We have developed the ChemicalTagger parser as a medium-depth, phrase-based semantic NLP tool for the language of chemical experiments. Tagging is based on a modular architecture and uses a combination of OSCAR, domain-specific regex and English taggers to identify parts-of-speech. The ANTLR grammar is used to structure this into tree-based phrases. Using a metric that allows for overlapping annotations, we achieved machine-annotator agreements of 88.9% for phrase recognition and 91.9% for phrase-type identification (*Action *names).

**Conclusions:**

It is possible parse to chemical experimental text using rule-based techniques in conjunction with a formal grammar parser. ChemicalTagger has been deployed for over 10,000 patents and has identified solvents from their linguistic context with >99.5% precision.

## Background

In many scientific disciplines, the primary method of communicating scientific results is in the form of a scientific paper or thesis which uses free flowing natural language combined with domain-specific terminology and numeric phrases. As such, they contain unstructured data, which is not identifiable by machines and not easily re-usable. Information providers have built businesses around the manual abstraction of unstructured data from the literature by human domain experts. Apart from the considerable labour cost and delay after the original publication, human abstraction is also a considerable source of error and data corruption.

A typical synthesis procedure taken from the organic chemistry literature, reads as follows: [1]

5-Cyclobutyl-2,3-dihydro-[1H]-2-benzazepine 82:

Potassium carbonate (0.63 g, 4.56 mmol) and thiophenol (0.19 g, 1.69 mmol) were added to the 2-nitrobenzene sulfonamide **50 **(0.50 g, 1.302 mmol) in N, N-dimethylformamide (33 cm^3 ^) at room temperature and the mixture was stirred for 16 h. Deionised water (50 cm^3 ^) was added and the aqueous phase was extracted with ethyl acetate (5 × 50 cm^3 ^). The organic extracts were dried (MgSO_4_) and concentrated under reduced pressure to give the title compound **82 **(0.259 g, 1.302 mmol, ca. 100%) as an oil used without further purification.

The example shown here shows highly stylized and formulaic language, which occurs in many disciplines, and is not just restricted to chemistry, and consists of:

**• Semi-structured documents**: Usually delimited by typographic conventions such as newlines and bold text, rather than formal markup.

**• Domain-specific entities**: Entities and terminology from different scientific domains.

**• Stock phrases**: *'X was added to a flask...'*.

**• Data phrases**: *'(0.259 g, 1.302 mmol, ca. 100%)'*.

Therefore, scientific papers are an attractive target for the development of machine processes for automatic information extraction. Text-mining uses NLP (Natural Language Processing) tools for the automatic discovery of previously unknown information from unstructured data. The information generated through text mining can be used for:

**• **The classification of documents (information retrieval).

**• **The determination of occurrence and co-occurrence of specific terms (indexing).

**• **The extraction of simple relationships.

**• **The systematic extraction of data from related studies.

**• **The generation of 'mashups' between different disciplines, such as the interactive Crystallography timemap developed by Ben O'Steen [2]. This visualisation shows authors of papers, geo-located onto a map and organised by the date of publication.

Text-mining in chemistry is not as prevalent as it is biology, and the tools are less developed. Text-mining in biology is often used for the automatic extraction of information about genes, proteins and their functional relationships from text documents [3-6]. The NLP tools in biology are also well developed, and we aim to create the equivalent in chemistry for part-of-speech taggers such as the GeniaTagger [7,8] as well as syntactic parsers such as Enju [9].

### Aims and Objectives

The aim of this paper is to show how text-mining has been achieved for the discipline of chemistry using our ChemicalTagger tool. Chemists not only produce a significant amount of data-rich scholarly communication artefacts, but have also adopted the highly formulaic style of writing outlined above. Consequently, these publications are an attractive target for automated data extraction. The sample paragraph quoted above will be used as an example throughout this paper, but it is stressed that the techniques reported here can be applied to much of science.

Previous work has concentrated on the identification and extraction of chemical entities from scientific papers [10-12], but did not address the extraction of the relationships linking these entities to both each other as well as to the document object from which they were extracted. The current work aims to address these issues using novel methods to extract information such as units, mixtures, amounts of substances and roles (such as solvents, reactants and products) as well as *Action *phrases using linguistic context. ChemicalTagger was initially developed in the context of physical science and has been designed to interoperate with bioscience tools and requirements as explored and presented at Dagstuhl 2008 [13]. The chemical literature considered in this work consists of journal articles, open-access theses and reports (*e.g*. company reports). Most of these documents have the general structure of 'Introduction', 'Materials and Methods', 'Experiments', 'Results', 'Discussion' and 'Summary and Conclusion'. This paper will focus on the experimental section, which usually consists of paragraphs such as the example shown above. The next section will demonstrate how relationships between entities can be extracted using our ChemicalTagger tool and stored in a machine-understandable format.

## Methods

ChemicalTagger is an open-source tool for tagging and parsing experimental sections in the chemistry literature. It takes a string of text as input and produces a structured XML document as output. The ChemicalTagger workflow can be divided into five main steps: text normalisation, tokenisation, tagging, phrase parsing and finally *Action *phrase identification. These steps will be described further below:

### Text Normalisation

Text normalisation is a preprocessing step that transforms the text into a format that is consistent for tagging. This involves removing nonprinting Unicode characters from text (these are the Unicode character set values 0 to 31, 127, 129, 141, 143, 144, and 157) and normalising the spacing between the words. To demonstrate, a sample phrase from the experimental paragraph above will be used:

'Potassium carbonate (0.63 g, 4.56 mmol) and thiophenol (0.19 g, 1.69 mmol) were added to the 2-nitrobenzene sulfonamide'

The text-normaliser first cleans the text of nonprinting characters such as non-breaking spaces, tabs and carriage returns. It then proceeds to formatting the spaces between alphanumeric and non-alphanumeric characters (*i.e*. commas, brackets, full stops...) within the sentence. In the sentence above, strings such as '(0.63 g,' and '(0.19 g,' could cause problems for the tagger as they are composed of four separate elements that have been combined, and consequently would be mistagged. The normaliser will break such strings down into their constituent parts *i.e *'(0.63 g,' and '(0.19 g,' respectively. Special care is taken with decimal points within numbers as well as brackets and commas within chemical names. After normalisation, the following text is produced:

'Potassium carbonate (0.63 g, 4.56 mmol) and thiophenol (0.19 g, 1.69 mmol) were added to the 2-nitrobenzene sulfonamide'

### Tokenisation

**Figure 1:**
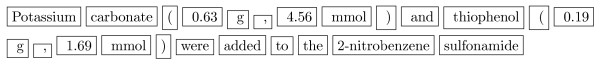
**Tokenisation**.

Tokenisation is the process of splitting a phrase into into a sequence of meaningful elements called tokens. A token can be made up of one or more words and is not necessarily alphanumeric (*e.g*. commas, exclamation marks, full stops etc...). Many different splitting patterns are conceivable in natural language processing and hence many different tokenisers exist, with the most common one being the whitespace tokeniser. An adapted whitespace tokeniser is used by ChemicalTagger, since chemical names, in particular, are fragile to common methods of tokenisation as they contain potential inter-token characters such as space, hyphens, brackets and commata. Running the tokeniser on the normalised sentence above produces the following tokens (Figure 1):

### Tagging

Tagging is the process of assigning grammatical roles to the tokens. ChemicalTagger uses a three-step cascading tagger. The first step involves running a chemical entity recogniser (OSCAR) on the tokens. ChemicalTagger then falls back on a customised regex tagger and then a parts-of-speech tagger for the tokens which have not been identified. The taggers will be discussed further below:

#### OSCAR-Tagger

OSCAR [11] is used for the recognition of chemical entities in text. OSCAR (Open Source Chemistry Analysis Routines) is an open source extensible system for the automated annotation of chemistry in scientific articles. It can be used to identify:

• Chemical names, including formulae and acronyms.

• Reaction names, such as *hydrolysis *and *Wolff-Kishner*.

• Ontology terms.

• Enzymes.

• Chemical prefixes and adjectives.

In addition, where possible, any chemical names detected will be annotated with structures derived either by lookup, or name-to-structure parsing using 'OPSIN' [14] or with identifiers from the ChEBI [15]('Chemical Entities of Biological Interest') ontology. The extracted information is stored in XML format. Identified chemical entities are marked up using the **ne **(named entity) tag. The tag has four attributes:

• **Id**: The id of the token within the document.

• **Surface**: The text that makes up the entity.

• **Type**: The chemical entity name, which can be either a chemical compound (CM), reaction name (RN), ontology term (ONT), chemical pre × (CPR), enzymes(ASE) or chemical adjective (CJ).

• **Confidence**: The confidence score associated with the identification of the entity, if the entity was identified using OSCAR's MEMM machine learning algorithm [16].

The XML output resulting from running the OSCAR parser on our sample text provides the following:

   <ne id='o1960'

      surface =' Potassium carbonate '

      type ='CM'

      confidence =

      '0.9448241038775597 ' >

      Potassium carbonate

   </ne >

   (0.63 g, 4.56 mmol) and

   <ne id='o1962'

      surface =' thiophenol '

      type ='CM'

      confidence =

      '0.9676694757597625' >

      thiophenol

   </ne >

were added to...

**Figure 2:**
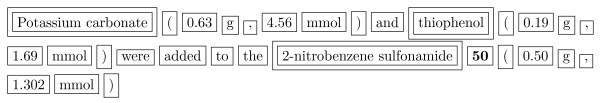
**OSCAR Tagging**.

At this stage, the chemical tokens have been successfully marked up (tokens denoted by single box and OSCAR-tagged tokens are shown in double boxes) (Figure 2):

#### Regex-Tagger

The Regex-Tagger is used to mark-up *chemistry-related *terms that are not recognised by OSCAR. These include nouns such as *solution *and *mixture *and verbs such as *quench *and *evaporate *that are specific to the chemistry domain. The Regex-tagger uses regular expressions that are stored in a *rules *file together with the customised tags. *chemistry-related *terms can include the following:

**• Boldface Numbers**: These numbers usually refer to a chemical in the experiment, such as the number **50 **in our example paragraph.

**• *Action*Verbs**: Verbs that refer to specific *Actions *in an experiment, such as adding, removing, dissolving etc...

**• Physical States**: The different aggregation states a chemical compound may have such as liquid (including oils), solid (including crystals) or gas.

**• Units**: Standardised quantities such as mmol, g and mL.

**Figure 3:**
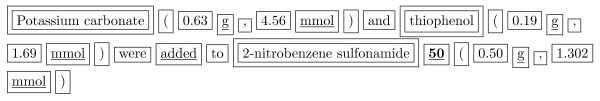
**Regex Tagging**.

This information is then passed to a regular expression tagger. Running this tagger on the sample phrase yields the following (tokens are denoted by single box, OSCAR-tagged tokens are in double boxes and regex-tagged tokens are underlined) (Figure 3):

#### English Parts-of-Speech Tagger

The final step of tagging involves marking up the general English language tokens. English parts-of-speech (POS) taggers are widely available and for the purposes of this work, the Penn Treebank is used. A treebank is a parsed text corpus (i.e. annotated with syntactic structure) that is used in corpus linguistics. The Penn Treebank [17] is commonly used for English parts-of-speech tagging and is made up of 4.5 million American English words. Typical tags include: [18]

**• NN **singular or mass noun

**• NNS **plural noun

**• VB **verb, base form

**• VBD **verb, past tense

**• CD **cardinal number (one, two, 2, etc.)

**• CC **Conjunctions (and, or, plus etc...)

**Figure 4:**
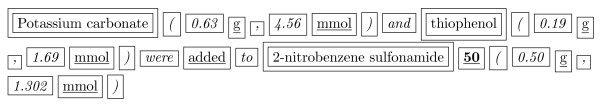
**English POS Tagging**.

This treebank is used within a parts-of-speech tagger, provided by OpenNLP. OpenNLP [19] is a suite of open source Java projects, data sets and tutorials supporting research and development in natural language processing. Running this tagger against the non-tagged text gives the following (tokens denoted by single box, OSCAR-tagged tokens are in double boxes, regex-tagged tokens are underlined and English POS-tagged tokens are in italics) (Figure 4):

At this stage, the text has been tokenised and tagged, it is now ready for parsing.

### Phrase Parsing

**Figure 5:**
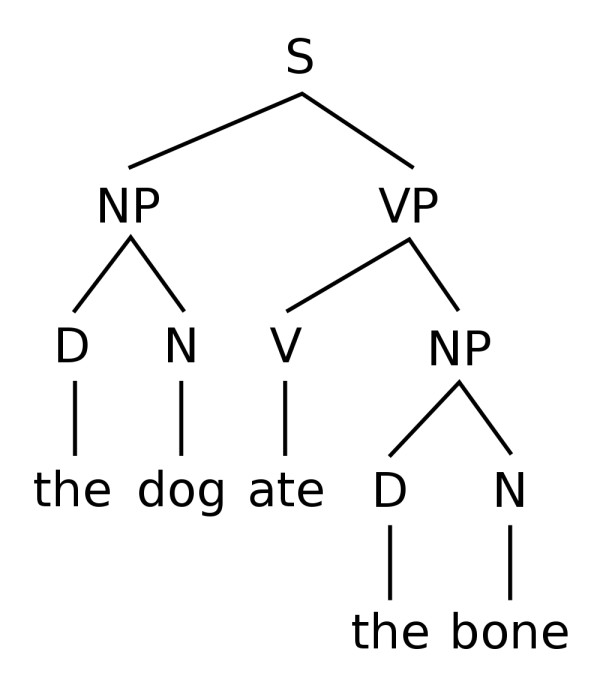
**Basic English Syntax Tree**. http://en.wikipedia.org/wiki/File:Basic_english_syntax_tree.svg.

Parsers build on tagged tokens to assign syntactical structure to text. The goal of phrase parsing in ChemicalTagger is to build the chemical equivalent of a Chomsky [20] tree structure of a sentence. Figure 5 is a syntactic tree model of a simple sentence.

In this tree model **S **is a sentence, **D **is a determiner, **N **a noun, **V **a verb, **NP **a noun phrase and **VP **a verb phrase.

In human discourse, sentences are parsed in multiple valid ways. However, the formualic structure of the chemical domain has a high probability for only one parse to be found. Therefore, a formal approach was decided on for phrase parsing so ChemicalTagger uses ANTLR [21]. ANTLR (ANother Tool for Language Recognition) is a parser generator that uses LL(*) parsing to automate the construction of language recognisers. It was designed to generate grammars for formal programming languages, but is applicable to any domain where an underlying implicit grammar exists. We believe that the type of language in our corpus can largely be described by a formal grammar, therefore ANTLR is used as a novel method for parsing phrases in chemical language.

LL(*) parsers are recursive descent parsers; they analyse input sequences by working their way down from the topmost non-terminal symbol until they reach a terminal node. In natural language these terminal nodes are the tokens. LL(*) parsers also use leftmost derivations and the symbols at each step are consumed from Left-to-Right, the '*' in LL(*) refers to the use of arbitrary lookahead to make decisions. According to Parr [21]:

LL(*)'s arbitrary lookahead is like bringing a trained monkey along in the maze. The monkey can race ahead of you down the various paths emanating from a fork. It looks for some simple word sequences from your [...]phrase that distinguish the paths. LL(*) represents a significant step forward in recognizer technology because it dramatically increases the number of acceptable grammars without incurring a large runtime speed penalty.

To demonstrate how ANTLR is used, a simplified version of ChemicalTagger's grammar is described below. For clarity, uppercase symbols represent terminals (symbols that can not be broken down into smaller constituents) while lowercase words represent non-terminals (symbols that can be broken down into smaller constituents).

The top-rule in our grammar is a *sentence *and it can be made up of a *nounphrase *and a *verbphrase*:

sentence: nounphrase verbphrase;

Using left derivation, the left non-terminal token *nounphrase *is selected. A *nounphrase *can be made up of a *determiner, adjective(s) *and *noun(s)*. A *noun *can include a *molecule *which in turn consists of an OSCAR recognised moiety followed by a *quantity*. A *quantity *consists of comma-separated numbers and units contained within brackets. This set of rules can be represented in ANTLR as follows:

nounphrase: determiner? adj* noun+;

noun: molecule+;

molecule: OSCARCM+ quantity?;

quantity: LRB mass COMMA molar RRB;

mass: CD NNMASS;

molar: CD NNMOLAR;

Once recursion down the *nounphrase *tree is completed, *verbphrase *is next. A *verbphrase *could consist of an *adverb, verb(s) *followed by a *prepphrase*. A *prepphrase *is made up of a preposition followed by a *nounphrase*.

**Figure 6:**
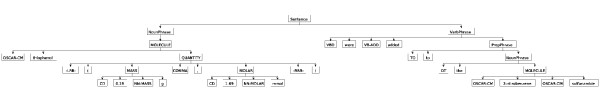
**AST Output of ANTLR Parse**.

Verbphrase: adv? verb+ prepphrase?;

verb: (VBD|VBADD);

prepphrase: TO nounphrase;

Running this grammar over the sample sentence produces the following output (Figure 6) in the form of an Abstract Syntax Tree (AST). The add-phrase shown here is only a simple example; more complex rules are defined to cover most of the grammar within the chemistry domain.

### Action Phrase Identification

The text has now been tagged and parsed, the next step is to assign roles to the parsed phrases. The roles, in this instance, refer to *Actions *carried out during a chemical synthesis (*e.g*. adding, dissolving, evaporating *etc*.). After surveying the literature in collaboration with domain experts, 21 different types of *Action *phrases were defined. The complete list of phrases can be found in Table 1.

**Table 1:** Phrases Recognised by ChemicalTagger

Phrase Name	Example
Add-Phrase	Benzoyl peroxide (85 mg) was **added **to the solution

Apparatus-Action	A 50-ml round-bottom flask **sealed **with a septum.

Concentrate-Phrase	The filtrate was **concentrated **under reduced pressure without heating.

Cool-Phrase	The reaction was then **cooled **to rt.

Degass-Phrase	The solution was **purged **with argon for 30 min.

Dissolve-Phrase	Salt was **dissolved **in water.

Dry-Phrase	The yellow product was **dried **under vacuum.

Extract-Phrase	the products were **extracted **with diethyl ether (3 × 100 ml).

Filter-Phrase	The solution was **filtered **through a short silica gel column.

Heat-Phrase	The mixture was **heated **under reflux for 8 h.

Partition-Phrase	The reaction mixture was **partitioned **between H2O (100 ml) and EtOAc (400 ml).

Precipitate-Phrase	**Precipitating **in methanol.

Purify-Phrase	The mixture was **purified **by column chromatography.

Quench-Phrase	The reaction was **quenched **with methanol.

Recover-Phrase	The precipitate was **recovered **by filtration.

Remove-Phrase	The solvent was **removed **under reduced pressure.

Stir-Phrase	The reaction mixture was **stirred **at room temperature for 16 h.

Synthesize-Phrase	**Synthesis **of aromatic polyethers by Scholl reaction.

Wait-Phrase	The mixture was **left **2d under stirring.

Wash-Phrase	The resin was **washed **with DMF.

Yield-Phrase	Chromatography **afforded **the alcohol 10 as a colourless oil (88 mg, 70%).

A postprocessing class was used to analyse the Abstract Syntax Tree, identify the *Action *phrases and output the tree to XML. Postprocessing our sample sentence gives the following XML output:

<Sentence >

    <ActionPhrase type = "Add">

        <NounPhrase >

            <MOLECULE >

                <OSCAR - CM >Potassium </OSCAR - CM >

                <OSCAR - CM >carbonate </OSCAR - CM >

                <QUANTITY >... </QUANTITY >

            </MOLECULE >

            <CC >and </CC >

            <MOLECULE >

                <OSCAR - CM >thiophenol </OSCAR - CM >

                <QUANTITY >... </QUANTITY >

            </MOLECULE >

        </NounPhrase >

        <VerbPhrase >

            <VBD >were </VBD >

            <VB - ADD >added </VB - ADD >

            <PrepPhrase >

                <TO >to </TO >

                <NounPhrase >

                    <MOLECULE >

                       <OSCAR - CM >2- nitrobenzene </OSCAR - CM >

                       <OSCAR - CM >sulfonamide </OSCAR - CM >

                    </MOLECULE >

                </NounPhrase >

            </PrepPhrase >

        </VerbPhrase >

        <STOP >. </STOP >

    </ActionPhrase >

</Sentence >

**Figure 7:**
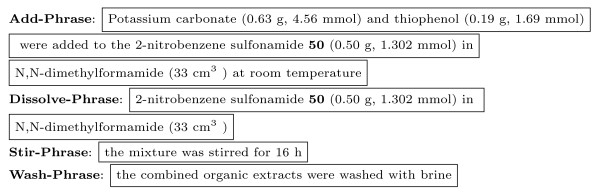
***Action *Phrase Markup**.

The following types of phrases can now be extracted from the preparation (Figure 7):

It is important to note, that the parser also extracts nested noun-phrases such as the **Dissolve-Phrase **found within the **Add-Phrase **as shown above.

#### Role Identification

This simple approach to *Action *phrase identification can yield good results. Other inferences can be made at this stage, such as the identification of 'roles'. Typical roles for chemical compounds are products, reactants and solvents. Using linguistic context such as *Action *names and their position in the text we are able to detect this. For example in the following Dissolve-Phrase:

*2-nitrobenzene sulfonamide ***50 **(0.50 g, 1.302 mmol) in **N, N-dimethylformamide (33 cm**^3^**) **and Wash-Phrase:

the combined organic extracts were washed with **brine**

it can be inferred that *N, N-dimethylformamide *and *brine *are solvents using cues such as their position after the preposition(underlined) and the type of Action phrase in which they are contained. The compound *2-nitrobenzene sulfonamide *may be classified as a reactant as a result of its location at the start of the text and the bold number **50 **following the compound. Bold numbers are commonly used as identifiers for reactants and products in organic chemistry literature. It can also be inferred that the product of this reaction is the compound *5-Cyclobutyl-2,3-dihydro-[1H]-2-benzazepine*, because of its location in the title

5-Cyclobutyl-2,3-dihydro-[1H]-2-benzazepine 82:

and the Yield-Phrase:

to give the title compound **82 **(0.259 g, 1.302 mmol, ca. 100%) as an oil.

The structure provided through ChemicalTagger facilitates these inferences (see the 'Architecture and Deployment' section).

### Output Representation

**Figure 8:**
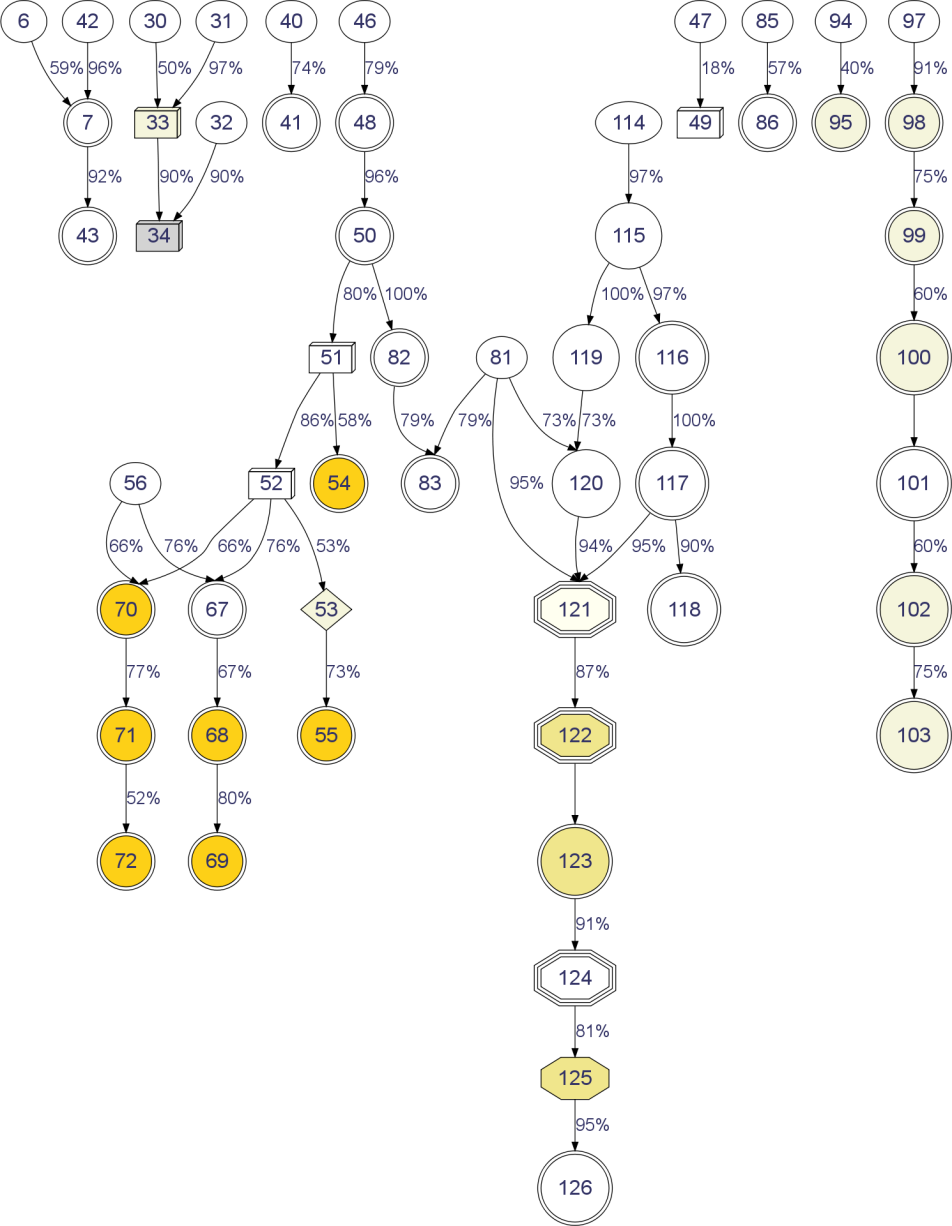
**Graph of Reaction Paths**.

The ultimate goal of ChemicalTagger is to create machine processable structured data from natural language. The parse trees and nodes need to be preserved and labelled to identify any phrase or language component within them. Output formats for this data include CML, XML and RDF. Storing information in a structured machine-processable format makes it readily available for querying and visualisation tools. For example, a query could be run to retrieve all reactions that use N, N-dimethylformamide as a solvent and 2-nitrobenzene sulfonamide as a reactant that have yields greater than 80%. This would be a useful tool for grouping together similar reactions. Structured information can also be visualised, Figure 8 shows one method of visualising extracted reaction paths.

In this figure, the numbers in the nodes refer to the products and reactants, the colours reflect the extracted information about the colour of the product and the shapes of the nodes refer to the aggregation state of the product:

• Ellipses: Unknown.

• 3D Boxes: Solid.

• Double Circles: Oil.

• Octagon: Gum.

• Triple Octagon: Foam.

• Diamond: Crystals or Needles.

As such, this graph provides a useful summary and a highly visual map of the chemistry reported in the paper -a document summary- and further analyses of this and graphs derived from other papers will open the door to the development of novel measures of document similarity (*e.g*. in terms of the chemical transformations reported in a corpus of synthesis papers).

### Architecture and Deployment

ChemicalTagger has been developed in a modular manner using the Java framework, making individual components such as tokenisers, vocabularies and phrase grammars easily replaceable. This facilitates the study of a wide range of chemical subdomains which vary in syntactic style, vocabulary and semantic abstraction. Moreover, it is possible to convert ChemicalTagger's output into CML [22] using a ChemicalTagger2CML converter. Thus, identified phrase-based chemistry such as solutions, reaction and procedures can converted into computable CML. This then allows for the construction of machine-processable synthesis information and searchable indices [23].

ChemicalTagger has been used in an initial study to index large numbers (*ca*. 10,000) of patents from the European Patent Office. Preliminary results of this work were presented at the Science Online meeting [24] where the methodology and deployment were demonstrated. The **Dissolve **phrases were extracted to determine what solvents were used. Although precise metrics were not used, the false positive rate (i.e. identification of a compound that was not a solvent) was very low (less than 0.5%) showing that ChemicalTagger greatly enhances the precision of identification of chemical compounds as well as providing the most likely role.

The modular structure of ChemicalTagger allows for adaption to general formulaic scientific language. Thus phrases that refer to conditions such as temperature (*at a temperature of *25°C), time (*left to equilibrate for 24 hours*), atmosphere (*under a nitrogen atmosphere*), and pressure (*caused by high pressure*) can be found in atmospheric or bioscience papers and we believe that ChemicalTagger will identify these phrases with high precision without further modification. We are intending to promote ChemicalTagger as an Open Source general scientific NLP tool. The source code is available at

https://bitbucket.org/lh359/chemicaltagger

and further information about ChemicalTagger can be found at

http://www-ucc.ch.cam.ac.uk/products/software/chemicaltagger

## Results and discussion

Evaluation was performed by preparing a corpus of experimental paragraphs from the chemical literature and conducting an inter-annotator study. The purpose of the inter-annotator agreement study is two-fold: evaluating the agreement between human annotators agree with each other and assessing the performance of ChemicalTagger against human annotators. Although chemistry is a relatively closed domain, writing styles vary and therefore such an assessment is necessary to get a clear picture of the quality of any machine extracted information.

### Corpus Assembly

The test corpus was assembled by carrying out searches for polymer synthesis related keywords in SciFinder Scholar [25]. The keywords were 'atom transfer radical polymerization', 'condensation polymerization' and 'anionic polymerization' and papers were chosen at random from a variety of journals. This was done in order to accommodate different writing styles and conventions used across the literature. 50 paragraphs from the experimental sections of these papers were used to create the corpus.

### Inter-Annotator Study

The study was carried out by four annotators, who are all trained chemists with formal backgrounds in different areas of chemistry. The annotators were provided with annotation guidelines, that can be found here [26]. The guidelines specify the structure of 21 different types of phrases that commonly occur in the chemistry literature and contain examples of annotated phrases from the experimental sections. The annotation process consisted of the chemists manually annotating 50 paragraphs from the test corpus and classifying the phrases according to the annotation guidelines. A point-and-click software tool was provided to facilitate annotation. After human annotation was completed, ChemicalTagger was run over the test corpus. Table 2 shows a the number of *Action *phrases marked up by all four annotators alongside the number of *Action *phrases marked up by ChemicalTagger.

**Table 2:** Number of Phrases Marked up by Annotators and ChemicalTagger

Phrase Name	Annotators' Markup	ChemicalTagger Markup
Add	46-49	47

ApparatusAction	18-23	21

Concentrate	10-11	11

Cool	23-28	24

Degass	19-29	22

Dissolve	29-34	30

Dry	36-40	39

Extract	11-12	10

Filter	21-26	20

Heat	15-26	17

Partition	2-7	3

Precipitate	15-20	13

Purify	25-32	26

Quench	16-16	16

Recover	0-9	9

Remove	18-21	30

Stir	33-37	34

Synthesize	50-66	46

Wait	2-8	14

Wash	25-26	25

Yield	35-39	36

**Total**	**490-527**	**493**

### Evaluation

Evaluation was performed by pair-wise comparison of the annotations (*i.e *Annotator A vs Annotator B); and the phrases as well as the *Actions *assigned to them were evaluated. Similarity of the annotations was measured using a Dice coefficient, a similarity matrix which is defined as:(1)

|*X*| and |*Y*| represent the annotations recognised by a pair of annotators. |*X *∩ *Y*| is the intersection of these annotations. The value of the similarity coefficient **s **therefore is twice the shared information over the combined set.

#### Identity of annotations

Previous work on annotations concentrated on named entities where strict rules for agreement between annotators. For example, in the sentence *We used sodium chloride solution *only the multiworded token *sodium chloride *would be allowed, while *sodium *and *sodium chloride solution *would both score negative. However, providing guidelines for measuring similarity between phrases is difficult. Conjunctions are problematic as are anaphora such as *Salt was dissolved in water and concentrated at *80°C. In this example there are two phrases, but 'and' is not part of either and its inclusion could score negatively. Alongside the identification of the extent of the phrase (which should be exact) the annotators were also asked to identify the types of phrase (in this case *dissolve *and *concentrate*). It is possible to match the extent correctly and misidentify the type, or vice versa. These considerations are critical to interpreting the performance of ChemicalTagger.

The *Action *types and phrases in the test corpus were evaluated separately. A string match was used to evaluate the *Action *types and a machine-annotator agreement of 91.9% was achieved (See Table 3).

**Table 3:** Action Name Agreement (%)

	Annotator1	Annotator2	Annotator3	Annotator4	ChemicalTagger
Annotator1	-	91.4	94.0	94.3	92.1

Annotator2	91.4	-	92.2	92.5	91.5

Annotator3	94.0	92.2	-	94.0	92.0

Annotator4	94.3	92.5	94.0	-	92.2

ChemicalTagger	92.1	91.5	92.0	92.2	-

					

**Machine-Annotator Agreement**	91.9				

**Inter-Annotator Agreement**	93.1				

Evaluating phrase similarity was more challenging as annotators can often get the sense of the markup without the exact extent. Exact string match produced a low inter-annotator agreement of 55.5% and machine-annotator agreement of 48.7%. For example, *and concentrated at *80°C and *concentrated at *80°C. do not match identically but have sufficient overlap that it is clear that the annotators were in agreement. Therefore a set of metric techniques based on string filtration were developed. The filter removed common stock words and tokens, such as preceeding adverbs and prepositions as well as '.', ',', ';', 'and', 'to', 'the' and 'a', from consideration. This improved the observed average Dice Coefficient considerably and achieved an inter-annotator agreement of 76.2% and a machine-annotator agreement of 60.4% (See Table 4).

**Table 4:** Filtered Phrase Agreement (%)

	Annotator1	Annotator2	Annotator3	Annotator4	ChemicalTagger
Annotator1	-	75.1	70.2	75.0	61.4

Annotator2	75.1	-	77.6	80.0	60.7

Annotator3	70.2	77.6	-	79.0	56.5

Annotator4	75.0	80.0	79.0	-	63.0

ChemicalTagger	61.4	60.7	56.5	63.0	-

					

**Machine-Annotator Agreement**	60.4				

**Inter-Annotator Agreement**	76.2				

#### Text Alignment

While filter matches improve the Dice coefficient considerably, this does not account for the ambiguity involved in defining and thus marking up the beginning and end of *Action *phrases. For example, the two *Action *phrases:

A 25 ml three-necked round-bottomed flask fitted with a dean-stark trap a condenser a nitrogen inlet/outlet

and

To a 25 ml three-necked round-bottomed flask fitted with a dean-stark trap a condenser a nitrogen inlet/outlet and magnetic stirrer

would, using the above metric, be treated as two different entities although they are essentially the same *Action *phrase. As such, a disagreement between two annotators is recorded if both have marked up slightly different beginnings and endings.

To solve this problem and get a true measure of the inter-annotator agreement, we have used the Needleman-Wunsch algorithm [27] to align and compare annotations by different annotators. The Needleman-Wunsch algorithm is a dynamic algorithm commonly used in bioinformatics to perform the global alignment of protein sequences. The algorithm aligns sequences by matching common characters and inserting spaces in unknown or non-matching locations. The alignment is performed by assigning scores for aligned characters in the form of a similarity matrix, with gaps being heavily penalised. An optimal alignment is then found. To illustrate how the algorithm works against annotations, consider the example in Table 5. The algorithm has detected that phrases 1 to 3 highlighted by both annotators match each other, while annotator A's phrase 4 does not match anything marked up by annotator B (and therefore gives a value of -1). Annotator A's fifth sentence was identified to correspond to annotator B's fourth sentence. The following matrix is produced by the alignment algorithm:

**Table 5:** Phrase Alignment Using the Needleman-Wunsch Algorithm

Annotator1	Annotator2
1. to a 25 ml three-necked round-bottomed flask fitted with a dean-stark trap, a condenser, and a nitrogen inlet/outlet and magnetic stirrer	1. a 25 ml three-necked round-bottomed flask fitted with a dean-stark trap, a condenser, and a nitrogen inlet/outlet

2.was subsequently sealed with a rubber septum	2. which was subsequently sealed with a rubber septum

3. stirring the reaction mixture overnight at room temperature	3. after stirring the reaction mixture overnight at room temperature

4. evaporation of the eluate	

5. afforded 8 as a white solid (2.63 g, 57% yield)	4. which then afforded 8 as a white solid (2.63 g, 57% yield)

[1, 2, 3, 4, 5]

[1, 2, 3,-1, 4]

A Dice coefficient was then calculated on the results of this alignment. Using this algorithm a machine-annotator agreement of 88.9% was achieved (see Table 6).

**Table 6:** Phrase Alignment Agreement(%)

	Annotator1	Annotator2	Annotator3	Annotator4	ChemicalTagger
Annotator1	-	90.2	89.2	91.1	88.4

Annotator2	90.2	-	90.8	91.6	89.8

Annotator3	89.2	90.8	-	91.6	87.2

Annotator4	91.1	91.6	91.6	-	90.2

ChemicalTagger	88.4	89.8	87.2	90.2	-

					

**Machine-Annotator Agreement**	88.9				

**Inter-Annotator Agreement**	90.8				

### Further Work

Current work investigates the use of chemical treebanks for recognising parts-of-speech tags as well as phrases. As mentioned earlier, a treebank is a parsed text corpus that is used in corpus linguistics for studying syntactic phenomena. It can also be used for training and testing parsers. Once parsed, a corpus will contain evidence of both frequency (how common different grammatical structures are in use) and coverage (the discovery of new, unanticipated, grammatical phenomena).

In life sciences, the Enju parser was adapted to biomedical domain by providing the GENIA treebank [9]. We aim to create an equivalent treebank for chemistry using an open-access corpus of paragraphs taken from the experimental sections of papers from the chemistry domain. This treebank will be produced semi-automatically by first running ChemicalTagger on the corpus and then manually correcting the mistagged nodes and trees. The treebank produced by this semi-automatic curation process will then be used as input for the development of a machine-learning-based parser for ChemicalTagger. An analysis of this parser's performance can then be carried out by evaluating its output against that of the ANTLR-based ChemicalTagger.

## Conclusions

We have shown that structured scientific data can be extracted from unstructured scientific literature using ChemicalTagger. We have also demonstrated that, using text mining and natural language processing tools, we can extract both chemical entities and the relationships between those entities, and make the resulting data available in a machine-processable format. We have shown that these graphs are useful for the generation of highly informative visualisations. While machine extraction can yield good results, it nevertheless remains an act of 'information archaeology' and as such necessarily imperfect. We therefore strongly urge, that the scientific community move towards an ethos where scientific data is published in semantic form and where both authors and publishers feel under an obligation to make this information openly available. Were this to happen on a significant scale, it would lead to a revolution where millions of chemical syntheses every year can be automatically analysed by machine, which in turn could lead to significant improvements in our ability to do science. Opportunities generated through the large-scale availability of semantic data include:

• Formal semantic verification of published information leading to higher quality information from authors, for reviewers and for technical processing.

• Greater understandability by readers (including machines).

• Automatic analysis of reaction conditions and results.

• Greater formal representation of chemical reactions.

We hope, however, that the extraction tools demonstrated here will have only a limited lifetime before they are replaced by semantic authoring.

### Copyright Implications

It is important to note that these extraction tools are restricted to the copyright associated with the data. Patents and Open Access (CC-BY) papers explicitly allow data extraction. Theses may depend on the copyright or explicit rights within the thesis. Most publishers of chemistry are not universally Open Access and we have engaged with them over several years trying to find a straightforward answer. The authors have raised this issue with both specific publishers (*e.g*. Elsevier, who publish Tetrahedron) and the STM Publisher's Association. Elsevier have referred this to their 'Universal Access' department and currently cannot say whether or not this is permitted. It has been agreed with STM publishers that bibliographic data is Open (CC-BY or CC0). There is no agreement, at the moment, on what data can be extracted.

## Competing interests

The authors declare that they have no competing interests.

## Authors' contributions

LH co-authored the paper, developed ChemicalTagger and evaluated its performance. DJ co-authored the paper and developed ChemicalTagger. NA co-authored the paper, setup the test corpus and co-authored the annotation guidelines. PMR co-authored the paper and was the principle investigator on the project.

## References

[B1] Bradshaw B Evans P Fletcher J Lee ATL Mwashimba PG Oehlrich D Thomas EJ Davies RH Allen BCP Broadley KJ Hamrouni A Escargueil C Synthesis of 5-hydroxy-2,3,4,5-tetrahydro-[1H]-2-benzazepin-4-ones: selective antagonists of muscarinic (M3) receptors Organic and Biomolecular Chemistry 2008 6 12 2138 2157 10.1039/b801206g 18528576

[B2] O'Steen B IUCr Crystal publication data http://benosteen.com/timemap/index last accessed: 09/02/11

[B3] Andrade MA Valencia A Automatic Annotation for Biological Sequences by Extraction of Keywords from MEDLINE Abstracts: Development of a Prototype System Proceedings of the 5th International Conference on Intelligent Systems for Molecular Biology 1997 AAAI Press 25 32 9322011

[B4] Cohen AM Hersh WR A survey of current work in biomedical text mining Briefings in bioinformatics 2005 6 57 71 10.1093/bib/6.1.57 15826357

[B5] Nenadić G Ananiadou S Mining semantically related terms from biomedical literature ACM Transactions on ALIP 2006 5 22 43

[B6] Rodriguez-Esteban R Methods in Biomedical Text Mining PhD thesis 2008 Columbia University

[B7] Tsuruoka Y Tateishi Y Kim JD Ohta T McNaught J Ananiadou S ichi Tsujii J Bozanis P, Houstis EN Developing a Robust Part-of-Speech Tagger for Biomedical Text Panhellenic Conference on Informatics Volume 3746 of Lecture Notes in Computer Science 2005 Springer 382 392

[B8] Genia Tagger http://www-tsujii.is.s.u-tokyo.ac.jp/GENIA/home/wiki.cgi?page=GENIA+Tagger last accessed: 28/11/10

[B9] Hara T Miyao Y Tsujii J Evaluating impact of re-training a lexical disambiguation model on domain adaptation of an HPSG parser Proceedings of the 10th International Conference on Parsing Technologies 2007 IWPT '07, Morristown, NJ, USA: Association for Computational Linguistics 11 22

[B10] Adams SE Goodman JM Kidd RJ McNaught AD Murray-Rust P Norton FR Townsend JA Waudby CA Experimental Data Checker: Better Information for Organic Chemists Organic and Biomolecular Chemistry 2004 2 3067 3070 10.1039/b411699m 15505708

[B11] Corbett P Murray-Rust P Berthold MR, Glen R, Fischer I High-Throughput Identification of Chemistry in Life Science Texts Computational Life Sciences II 2006 Springer Berlin/Heidelberg 107 118

[B12] Townsend JA Downing J Murray-Rust P CHIC - Converting Hamburgers into Cows Fifth IEEE International Conference on e-Science: 9-11 December 2009; Oxford, IEEE 2009 337 343

[B13] Ashburner M Leser U Rebholz-Schuhmann D (Eds) Ontologies and Text Mining for Life Sciences: Current Status and Future Perspectives Dagstuhl Seminar Proceedings 2008 Dagstuhl, Germany: Schloss Dagstuhl - Leibniz-Zentrum fuer Informatik, Germany

[B14] Lowe DM Corbett PT Murray-Rust P Glen RC Chemical Name to Structure: OPSIN, an Open Source Solution J Chem Inf Model 2011 51 3 739 53 10.1021/ci100384d 21384929

[B15] Degtyarenko K de Matos P Ennis M Hastings J Zbinden M McNaught A Alcántara R Darsow M Guedj M Ashburner M ChEBI: a database and ontology for chemical entities of biological interest Nucleic Acids Res 36 D344 D350 10.1093/nar/gkm791 PMC2238832 17932057

[B16] Corbett P Copestake A Cascaded classifiers for confidence-based chemical named entity recognition BMC Bioinformatics 2008 9 Suppl 11 S4 10.1186/1471-2105-9-S11-S4 19025690 PMC2586753

[B17] The Penn Treebank Project http://www.cis.upenn.edu/~treebank/last accessed: 28/11/10

[B18] Penn Treebank TagSet http://www.ling.upenn.edu/courses/Fall_2003/ling001/penn_treebank_pos.html last accessed: 07/04/11

[B19] Open NLP Website http://incubator.apache.org/opennlp/last accessed: 07/04/11

[B20] Chomsky N Syntactic Structures 1957 The Hague: Mouton

[B21] Parr T The Definitive ANTLR Reference: Building Domain-Specific Languages 2007 Raleigh: The Pragmatic Bookshelf

[B22] Murray-Rust P Rzepa HS Wright M Zara S A universal approach to web-based chemistry using XML and CML Chemical Communications 2000 1471 1472

[B23] Holliday GL Murray-Rust P Rzepa HS Chemical markup, XML, and the world wide web. 6. CMLReact, an XML vocabulary for chemical reactions J Chem Inf Model 2006 46 145 157 10.1021/ci0502698 16426051

[B24] Science Online London 2010 http://scienceonlinelondon.wikidot.com/topics:green-chain-reaction last accessed: 28/11/10

[B25] Scifinder Scholar http://www.cas.org/products/sfacad/index.html last accessed: 28/11/10

[B26] Annotation Guidelines for Marking up Chemistry Phrases http://wwmm.ch.cam.ac.uk/wikis/wwmm/index.php/Image:Guidelines.pdf last accessed: 28/11/10

[B27] Needleman SB Wunsch CD A general method applicable to the search for similarities in the amino acid sequence of two proteins Journal of molecular biology 1970 48 3 443 453 10.1016/0022-2836(70)90057-4 5420325

